# The current use of proteomics and metabolomics in glomerulonephritis: a systematic literature review

**DOI:** 10.1007/s40620-024-01923-w

**Published:** 2024-04-30

**Authors:** Elin Davies, Andrew Chetwynd, Garry McDowell, Anirudh Rao, Louise Oni

**Affiliations:** 1 Department of Women’s and Children’s Health, Institute of Life Course and Medical Sciences, University of Liverpool, Liverpool, UK; 2 Department of Nephrology, Liverpool University Hospitals NHS Foundation Trust, Liverpool, UK; 3 Centre for Proteome Research, Department of Biochemistry, Cell and Systems Biology, Institute of Systems, Molecular and Integrative Biology, University of Liverpool, Liverpool, UK; 4 Liverpool Centre for Cardiovascular Science, University of Liverpool, Liverpool John Moores University and Liverpool Heart and Chest Hospital, Liverpool, UK; 5 Clinical Directorate, Institute of Life Course and Medical Sciences, University of Liverpool, Liverpool, UK; 6 School of Pharmacy and Biomolecular Sciences, Liverpool John Moores University, Liverpool, UK; 7 Research Laboratory, Liverpool Heart and Chest Hospital, Liverpool, UK; 8 Department of Paediatric Nephrology, Alder Hey Children’s, NHS Foundation Trust Hospital, Eaton Road, Liverpool, UK

**Keywords:** Glomerulonephritis, Metabolomics, Proteomics, Biomarker

## Abstract

**Background:**

Glomerulonephritis inherently leads to the development of chronic kidney disease. It is the second most common diagnosis in patients requiring renal replacement therapy in the United Kingdom. Metabolomics and proteomics can characterise, identify and quantify an individual’s protein and metabolite make-up. These techniques have been optimised and can be performed on samples including kidney tissue, blood and urine. Utilising omic techniques in nephrology can uncover disease pathophysiology and transform the diagnostics and treatment options for glomerulonephritis.

**Objectives:**

To evaluate the utility of metabolomics and proteomics using mass spectrometry and nuclear magnetic resonance in glomerulonephritis.

**Methods:**

The systematic review was registered on PROSPERO (CRD42023442092). Standard and extensive Cochrane search methods were used. The latest search date was March 2023. Participants were of any age with a histological diagnosis of glomerulonephritis. Descriptive analysis was performed, and data presented in tabular form. An area under the curve or p-value was presented for potential biomarkers discovered.

**Results:**

Twenty-seven studies were included (metabolomics (*n* = 9)), and (proteomics (*n* = 18)) with 1818 participants. The samples analysed were urine (*n* = 19) blood (*n* = 4) and biopsy (*n* = 6). The typical outcome themes were potential biomarkers, disease phenotype, risk of progression and treatment response.

**Conclusion:**

This review shows the potential of metabolomic and proteomic analysis to discover new disease biomarkers that may influence diagnostics and disease management. Further larger-scale research is required to establish the validity of the study outcomes, including the several proposed biomarkers.

**Graphical abstract:**

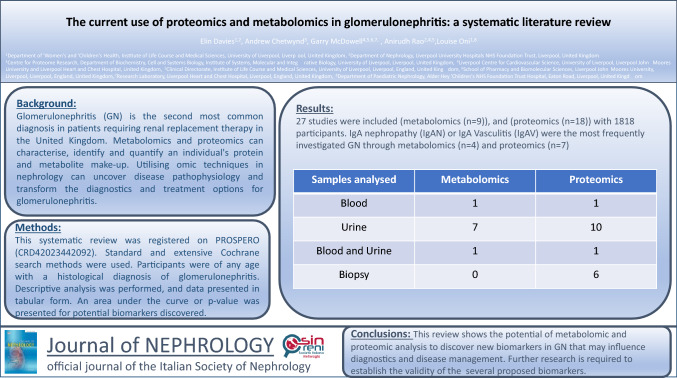

## Introduction

Kidney disease is increasingly becoming a significant worldwide health burden [1]. The global all-age chronic kidney disease (CKD) mortality increased by 41.5% between 1990 and 2019 [2, 3]. There is a growing, unified acknowledgement of an unmet need in identifying patients with CKD and managing risk factors for disease progression [4–6].

Glomerulonephritis (GN) is a leading cause of CKD, with CKD prevalence continuing to increase [7]. In the United Kingdom, in paediatric and adult populations, GN is the second most common primary renal diagnosis in those commencing kidney replacement therapy [8]. Glomerulonephritis presents treatment challenges due to the many intricate immunopathogenic processes that remain to be fully characterised. At present, a kidney biopsy is required to identify histopathological lesions and patterns that correlate with a specific GN diagnosis. With advances in our appreciation of the heterogeneity of GN, it is believed that confirmatory histology does not uncover the immunopathogenesis of the active inflammatory process at play. Further, there remains a pressing need to expand our use of immunomodulatory drugs and discover new drugs as, to date, there is an ongoing dependence on glucocorticoids as the mainstay of treatment, exposing patients to their long-term side effects [9–11].

To strengthen our clinical diagnosis, it is imperative that we are confident of the molecular architecture of a diseased state. The “omics” refers to a group of scientific disciplines aiming to generate large quantities of data by characterising different layers of the biochemical composition of a biological system. The most utilised of these sciences are genomics, transcriptomics, proteomics and metabolomics. These approaches consolidate our understanding of disease pathogenesis and phenotypes and are becoming integral to translational precision medicine, with biomarker discovery now frequently based on omic data [12–14]. Through omics analysis, samples can be comprehensively characterised, and these results can be interpreted alongside the clinical data [15, 16].

In the case of kidney disease, both metabolomics and proteomics techniques have become well established, allowing blood, urine and tissue samples to be analysed [17]. Untargeted proteomics (also known as bottom-up or shotgun proteomics) analyses the protein composition of a biological system [18]. In many instances, these proteins can be modified with other chemical classes which impact the structure, function and stability of proteins and include; phosphate groups or carbohydrates known as glycans, the latter of which are well established and contribute to kidney disease [19].

Metabolomics refers to the characterisation of “small molecules” typically < 1000 Da in size that encompass key metabolic components such as amino acids, steroids, bile acids and organic acids, which comprise enzymatic substrates, cofactors and products [20]. Metabolomics has been widely applied to GN [21], acute kidney injury (AKI) [22] and the development of kidney cancer [23]. Lipidomics is a sub-category of the metabolome, characterising all lipids within a biofluid or tissue, such as phospholipids, triacylglycerides, eicosanoids and fatty acids. Lipidome dysregulation, in particular, has been linked to CKD and cardiovascular risk [24, 25]. It is becoming increasingly acknowledged that the wealth of information to be discovered through multi-omic analysis, including proteomics and metabolomics, can lead to the development of precision medicine in CKD [26, 27].

The aim of this study was to perform a systematic literature review to summarise the current application of proteomic and metabolomic techniques for GN to identify strengths and areas of unmet need.

## Methods

This systematic review was registered in PROSPERO (CRD42023442092). The inclusion criteria were patients of any age, sex or ethnicity who had a histological or genetic diagnosis of GN as per the Kidney Disease: Improving global outcomes (KDIGO) criteria [28]. The methods included were; the use of one of the three most frequently applied analytical techniques: liquid chromatography–mass spectrometry; gas chromatography–mass spectrometry or proton nuclear magnetic resonance untargeted metabolomic or proteomic analysis; and any human biofluid or tissue. Studies based on therapeutic drug monitoring were excluded.

The PICO framework for the systematic review was:

*Population*: Patients of any age, sex or ethnicity who had a histological or genetic diagnosis of GN as per the KDIGO criteria [28]

*Intervention*: Untargeted metabolomics (including lipidomics) or proteomic analysis.

*Comparator*: Currently adopted lab techniques in clinical practice.

*Outcome*: Discovery of clinically relevant results that can change current practice.

Three online databases were searched on the 14th March, 2023: Cochrane, Ovid and Scopus.

The study designs included were meta-analyses, randomised control trials, cohort studies, case–control studies, cross-sectional studies and case series (n > 5). The filters applied to the search tool were; an original publication date between 2013 and 2023 (allowing for an inclusion period of 10 years), accessible in full text through the University of Liverpool, an abstract available in English with sufficient data for extraction. Studies that identified exogenous metabolites (such as those associated with ingested food products or drugs) as biomarkers were excluded alongside secondary data and animal studies. The reference lists of relevant literature were hand-searched to identify any additional eligible studies.

The search terms applied to the databases were;

(Glomerulonephritis) 0R (IgA nephropathy) OR (membranous nephropathy) OR (fsgs) OR (focal segmental glomerulosclerosis) OR (Nephrotic syndrome) OR (minimal change disease) OR (Lupus nephritis) OR (Membranoproliferative glomerulonephritis) OR (mpgn) OR (ANCA-associated vasculitis) OR (Antineutrophil cytoplasmic antibody associated vasculitis) OR (microscopic polyangiitis) OR (Mpa) OR (eosinophilic granulomatosis with polyangiitis) OR (egpa) OR (wegener's granulomatosis) OR (anti-GBM antibody) OR (anti-glomerular basement membrane) OR (goodpastures) **AND (**Omic) OR (Proteomics) OR (Metabolomics) OR (Lipidomics) OR (Mass spectrometry) OR (GC–MS) OR (NMR) OR (LC–MS).

### Selection process

Four reviewers completed title screening independently: AC, ED, LO, and AR. Abstract screening and full text screening was completed by two reviewers (AC and ED). At every level of review any conflicts were discussed and subsequently resolved. Duplicate results were screened electronically by Rayaan software, and any further remaining duplicates were manually removed after cross-checking. The Critical Appraisal Skills Programme (CASP), Cohort study checklist was applied to each included study to evaluate the quality of the study to determine the risk of bias [29].

### Data collection and analysis

Descriptive analysis was applied to the data collected from the included studies and presented in tabular form. The data outcomes extracted from each study were; first named author, country of study, publication year, study design, subtype GN, cohort demographics, sample analysed, analytical technique utilised and key outcomes. Area under curve (AUC) or p-value was presented for those studies that identified potential biomarkers or statistically significant molecular discoveries.

Sex distribution was converted to a percentage of males. The average age was calculated from those studies which provided complete demographic data. Study demographics were split into the named GN subtype, disease control or healthy control where applicable. Incomplete data values were recorded as NA.

The study was split into two groups depending on the omic analysis utilised: metabolomics (including lipidomics) and proteomics.

## Results

### Data extraction

**Fig. 1:**
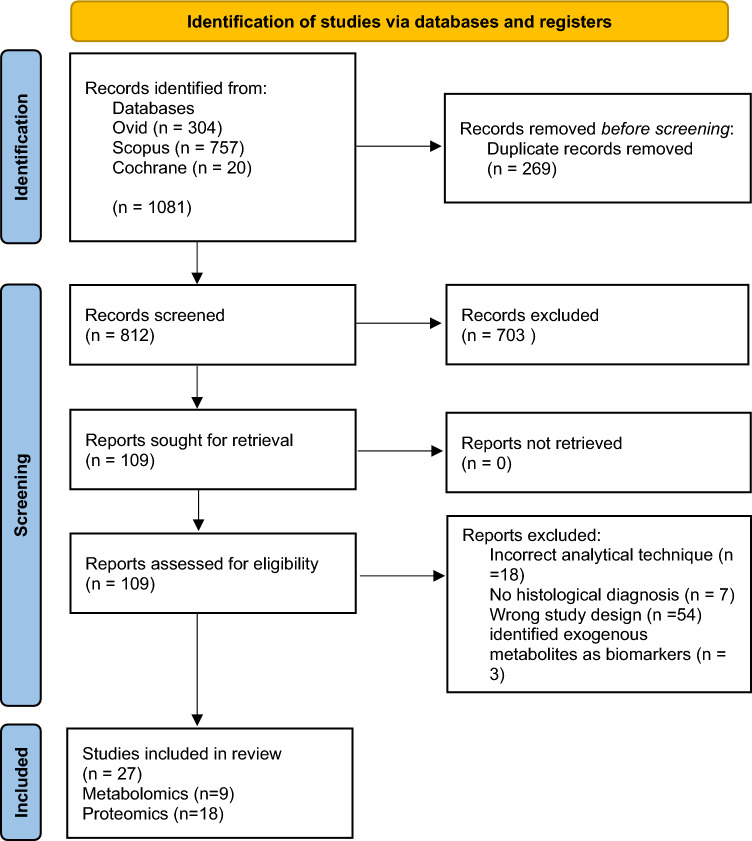
A flow diagram of the screening process. Literature search performed on four databases returned a total of 1081 papers. Following removal of duplicates, 812 papers were screened. After screening by an initial and a second independent researcher, a total of 27 studies were included in the systematic review

An online database search was completed in March 2023 and yielded 1081 papers. A total of 269 duplicates were identified and removed. The remaining 812 records were screened by abstract and a subsequent 109 were included for full-text review. The final number of papers included for review was 27. No further papers were included from screening reference lists. The process of article selection is shown in Fig. 1.

### Quality assessment

The CASP checklist was applied to all included studies. The checklist highlighted the risk of bias in those studies without a control cohort and that prior exposure to immunosuppression was an important confounding factor that was not accounted for in some studies.

### Metabolomics and lipidomics

A total of 9 included studies were based on metabolomics (*n* = 8) and lipidomics (*n* = 1) and used different analytical platforms: proton nuclear magnetic resonance (*n* = 6), gas chromatography–mass spectrometry *n* = (2), liquid chromatography–mass spectrometry (*n* = 2). The total population cohort included 1,196 patients (Average cohort size 133, range 13–497), of whom 287 (24%) were healthy controls. The overall sex distribution included 45% males with an average age of 38 years (range 6–50). The samples analysed were urine (*n* = 7), blood (*n* = 1) and both blood and urine samples (*n* = 1). IgA nephropathy (IgAN) and IgA Vasculitis (IgAV) were the most frequently investigated GN (*n* = 4), followed by focal segmental glomerulosclerosis (FSGS) (*n* = 3) membranous nephropathy (MN) (*n* = 2), lupus nephritis (LN) (n = 2), minimal change disease (MCD) (*n* = 2).

Two studies investigating IgAN, did not include a healthy cohort of patients for comparison. All studies were cross-sectional cohort studies.

A summary of the key results from the studies using metabolomics and lipidomics in GN is presented in Table 1.

**Table 1:** Metabolomics and lipidomic study methodology and results

Metabolomics and lipidomics													
Author Country Year	Study Design	GN	Cohort		Demographics				Sample type	Analytical technique	Outcome		
			Diagnosis	Number (n)	Age (years)		Male sex %				Total number of differentiating metabolites	Proposed biomarker and function	Area under curve
Zhang et al*.* [30] China 2021	Metabolomics	IgA Vasculitis (IgAV) IgAN	D IgAV	46	42		59		Blood Urine	LC–MS	38 in serum 50 in urine	Choline and cis-vaccenic acid Differentiate between IgAV and IgAN	0.927 serum 0.724 urine
			D IgAN	44	32		45						
			HC										
Kalantari et al*.* [31] Iran 2017	Metabolomics	IgAN	D	13	33		85		Urine	H^1^NMR	Not specified	Most relevant pathways in severity of IgAN phenylalanine metabolism', 'tyrosine metabolism', 'phenylalanine, tyrosine and tryptophan biosynthesis' nitrogen metabolism The most significant pathway that correlated with severity of IgAN 'phenylalanine metabolism' six metabolites: L-phenylalanine, L-tyrosine, trans-cinnamic acid, hydrocinnamic acid, 3-hydroxyphenylacetic acid fumaric acid *p* < 0.0001	Not calculated
			DC										
			HC										
Park et al*.* [32] Korea 2021	Metabolomics	IgAN	D	201	43		56		Urine	H^1^NMR Validated with LC–MS	Total number not specified 15 metabolites were significantly higher in the IgAN group than in HC: alanine, betaine, choline, creatinine, dimethylamine, formate, glycine, isoleucine, lactate, leucine, pyruvate, threonine, trimethylamine N-oxide, valine and t-methylhistidine	Model using clinical variables and urine metabolites including glycine; Age, sex, baseline eGFR^a^, mean arterial pressure, uPCR^b^ Diagnostic of IgAN	0.931
			DC	160									
			HC	136									
Guleria et al*.* [33] India 2016	Metabolomics	LN SLE (Systemic Lupus Erythematous)	D	40	29		8		Blood	H^1^NMR	Not specified	Lipids, lipoproteins and acetate Distinguish LN from SLE	> 0.95
			DC SLE	22	32		0						
			HC	30	28		17						
Kalantari et al*.* [34] Iran 2019	Metabolomics	LN	D	14	38		21		Urine	H^1^NMR	Total number not specified 13 metabolic differences between (LN) and (HC) and between LN and SLE 4-Methylcatechol 3,4-Dihydroxyphenylacetaldehyde 2,2-Dimethylsucssinic acid Beta-alanine Nicotinamide ribotide Nicotinamide Nicotinamide adenine dinucleotide Nicotinic acid Guanosine triphosphate Epi-coprostanol Pyridoxine Hippuric acid Anthranilic acid	beta-alanine, 2,2-dimethylsuccinic acid, 3,4-Dihydroxyphenylacetaldehyde diagnostic panel for LN	0.89
			DC SLE	10	41		20					2,2-dimethylsuccinic acid discriminator between LN and SLE	0.88
			HC	11	39		36						
			DC										
			HC	33	40		40						
Taherkhani et al*.*[35] Iran 2019	Lipidomics	Idiopathic MN	D	79		39		63	Urine	H^1^NMR GC–MS	Not specified	α-hydroxybutyric acid, 3,4-Dihydroxymandelic acid, 5α-cholestanone, 2-Hydroxyglutaric acid lactone, nicotinamide, epicoprostanol, and palmitic acid A panel composed of seven metabolites the best diagnostic predictors of MN	1
			DC	83									
			HC	53									
Hao et al*.* [36] China 2013	Metabolomics	FSGS	D	25		40		52	Urine	H^1^NMR	Not specified	glucose, dimethylamine and trimethylamine increased compared with healthy controls, while pyruvate, valine, hippurate, isoleucine, phenylacetylglycine, citrate, tyrosine, 3-methylhistidine and β-hydroxyisovalerate decreased Lower urine N-methylnicotinamide levels compared with other glomerulopathies	Not applicable
			DC	64		38		47					
			HC	35		41		49					
Erkan et al*.* [37] USA 2016	Lipidomics	FSGS MCD	D FSGS	8		14		88	Urine	LC–MS	Not specified	In FSGS cohort: Increased urinary concentration of fatty acid and lysophosphatidylcholines and a decrease in urinary concentration of phosphatidylcholine	Not calculated
			D MCD	10		6		50				Low eGFR correlated with lower urinary acylcarnitine C12:0 concentration (p < 0.05) Subgroup analysis of FSGS split into egfr	Not calculated
			HC	10		10		60					
Lee et al*.* [38] South Korea 2016	Metabolomics	MCD FSGS MN	MCD	30		49		61	Urine	GC–MS	33	panel of 5 metabolites; citric acid, pyruvic acid, fructose, ethanolamine, and cysteine Differentiate Nephrotic syndrome pathology	0.889 to 0.951
			FSGS	30									
			MN	30									
			HC	12									

^a^eGFR—estimated glomerular filtration rate, ^b^ uPCR—Urine protein creatinine ratio add missing abbreviaitons

### Potential diagnostic biomarkers

Four papers identified potential diagnostic biomarkers for specific GN subtypes. The Taherkhani et al. [35] study included 79 MN patients, 83 disease controls and 53 healthy controls with an average age of 39 years. A panel of seven lipid metabolites in urine was identified that could differentiate idiopathic MN from healthy controls and disease controls with an AUC 1.0. This study used two different analytical techniques to analyse samples, gas chromatography–mass spectrometry and proton nuclear magnetic resonance. The proposed metabolites panel reflected those of significance across both analytical strategies. Park et al.[32], in a study of 201 IgAN compared with 160 disease controls and 136 healthy controls with an average age of 43 years, analysed urine samples using proton nuclear magnetic resonance and validated the results with liquid chromatography–mass spectrometry. A model was developed using identified biomarkers alongside demographics (age and sex), kidney parameters (estimated glomerular filtration rate (eGFR), urine protein: creatinine ratio), and mean arterial pressure. This model was diagnostic of IgAN with AUC 0.931.

### Disease phenotype and risk of progression

Two studies without healthy control cohorts analysed samples from IgAN patients. Zhang et al. [30] identified two lipid-related molecules, Choline and Cis-vaccenic acid, present in both serum and urine, that could distinguish IgAV from IgAN. Forty-six IgAV and 44 IgAN patients were included, with an average age of 37 years. A panel of choline and cis-vaccenic acid gave an AUC of 0.927 in serum and 0.7243 in urine, which could distinguish between disease phenotypes.

Kalantari et al. [31] studied a cohort of 13 IgA patients with an average age of 33 years that were separated into mild and severe groups depending on Oxford biopsy classification. The aim was to establish any urinary biomarkers that could correlate with the histological classification of disease. Nine metabolites were positively correlated with proteinuria, and three were negatively correlated with proteinuria. The results also identified that phenylalanine metabolism was a significant metabolic pathway that was altered and correlated with disease progression.

Distinguishing between systemic lupus erythematosus (SLE) and LN and a healthy control using lipidomic analysis of serum samples was investigated by Guleria et al. [33], identifying an altered lipid metabolome that could identify LN activity. Guleria et al. [33] studied 22 SLE, 40 LN and 30 healthy controls with an average age of 30 years. Elevated serum levels of low-density/very low-density lipoproteins (triglyceride and fatty acid) and decreased serum levels of acetate were apparent in LN. Data analysis included investigating the correlation between the discriminatory serum metabolites and SLE disease activity index (SLEDAI) for the SLE group, but no significant correlation was observed.

### Proteomics

A total of 18 studies were included, all studies utilised liquid chromatography–mass spectrometry. The cohort included 622 patients (average cohort size 35, range 10–103) as GN or disease controls and 135 healthy controls. The average sex distribution across all study cohorts was 55% male, with an average age of 32 years (Range 4–60). The samples analysed were urine (*n* = 11), biopsy (*n* = 6) and blood (*n* = 2); one study analysed both blood and urine samples. IgAN was the most frequently investigated GN (*n* = 7), followed by MN (*n* = 4), LN (*n* = 4), FSGS (*n* = 5), and MCD (*n* = 1).

A total of 15 studies were cross-sectional cohort studies alongside three longitudinal studies aimed to identify responses to treatment. Seven studies had a healthy control cohort. Three studies did not have any demographic data. Two studies exclusively investigated a paediatric population with a cohort size of 61 and 18, respectively.

A summary of the key results from the proteomic studies in GN is presented in Table 2.

**Table 2:** Proteomics study methodology and results

Proteomics										
Author Country Year	Study Design	GN	Cohort		Demographics		Sample	Analytical Technique	Outcome	
			Diagnosis	Number	Age (years)	Male sex %			Number proteins identified	Biomarker or altered pathways
Samavat et al. [39] Iran 2015	Cross sectional	IgAN	D	13	33	85	Urine	LC–MS	493	13 proteins were upregulated, and 33 proteins were downregulated in IgAN
			DC							
			HC	8	35	75				
Xue et al.[40] China 2023	Cross sectional	IgAN	D	60	No data	No data	Blood	LC–MS	512 37 proteins > twofold change in three ckd stages when compared with HC	18 significantly changed proteins
			DC							
			HC	43						
Fang et al. [41] China 2021	Cross sectional	IgAN	D	19	9	84	Urine	LC–MS	1830 IgAN 276 urinary proteins differentially expressed IgAV 125 urinary proteins differentially expressed	Alpha-1B-glycoprotein (A1BG) and Afamin raised compared to HC in IgAN and IgAV *p* < 0.05
			DC	19	9	78				
			HC							
Kalantari et al. [42] Iran 2013	Cross sectional	IgAN	D	13	33	85	Urine	LC–MS	232 62 proteins > 1.5-fold change	Impairment of Extra Cellular Matrix (ECM)-Receptor Interaction pathways Activation of complement and coagulation pathway in progression of IgA nephropathy
			DC							
			HC							
Mucha et al. [43] Poland 2014	Cross sectional	IgAN	D	30	40	50	Urine	LC–MS	1238	18 proteins differentiated IgAN v HC p < 0.05
			DC							
			HC	30	39	50				
Paunas et al. [44] Norway 2022	Cross sectional	IgAN	D Progressive IgA	9	28	57	Biopsy	LC–MS	2564 150 proteins were differentially abundant between progressive and non-progressive IgAN *p* < 0.05	Periostin biomarker of progression AUC 0.91
			D Non-progressive	18	33	83				
			HC							
Kawata et al. [45] Japan 2020	Cross sectional	IgAN MN	D IgAN	5	37	40	Biopsy	LC–MS	483	Immunoglobulins and complement elevated compared to HC
			D MN	5	54	20				
			HC	5	60	20				
Turnier et al [46] USA 2019	Cross sectional	LN	D	61	16	29	Urine	LC–MS	112	α1-antichymotrypsin (SERPINA3 gene) levels also significantly increased with higher histological LN activity P = 0.03 SERPINA3 moderate positive association with disease severity p = 0.005
			DC							
			HC							
Chen et al [47] China 2022	Cross sectional	Lupus	D Membranous LN	11	31	19	Biopsy	LC–MS	5112 proteins 16 proteins exclusively found in membranous LN 85 proteins were exclusively found in the proliferative group	None identified
			DC Proliferative LN	12	33	25				
			HC							
Ghasemi et al. [48] Iran 2021	Longitudinal	LN 11 completed follow-up	D	19	34	16	Urine Blood	LC–MS	Serum 326 proteins Urine 1381 proteins	Biomarkers for treatment response: Twenty plasma proteins and ten urine proteins identified as potential
			DC							
			HC							
Mao et al. [49] China 2021	Cross sectional	LN	D	10	33	20	Biopsy	LC–MS	4364 proteins 72 differentially expressed	High expression of renal NEU1(enzyme) was identified as an independent risk factor for renal prognosis by multivariate Cox regression analysis (HR, 6.462 (95% CI 1.025 to 40.732
			DC							
			HC							
Pang et al. [50] China 2018	Cross sectional	MN	D PLA2R^a^ POSITIVE	32	52	72	Urine	LC–MS	249 proteins identified	Overexcretion of alpha-1-antitrypsin (A1AT) and afamin (AFM) Immunisation and coagulation were predominantly involved
			D PLA2R NEGATIVE	31	43	52				
			HC	32	43	43				
Li et al. [51] China 2022	Cross sectional	MN	D	16	No data		Biopsy	LC–MS	4529 proteins 3241 phosphorylated sites identified in 1704 proteins	Phosphoproteins likely important signalling molecules in development of MN
			DC	16						
			HC							
Rood et al. [52] Netherlands 2015	Cross sectional	MN FSGS	D	5	No data		Urine	LC–MS	245 proteins identified	LIMP-2 peptides *p* < 0.01 in MN
			DC	5						
			HC	5						
Kalantari et al. [53] Iran 2014	Longitudinal	FSGS	D Steroid sensitive	6	37	60	Urine	LC–MS	368 proteins 21 protein candidates identified	the most drastic fold change exhibited in steroid sensitive group apolipoprotein A-I (APOA-1) increased and Matrix-remodelling protein 8 (MXRA8) decreased
			D Steroid resistant	4						
			HC							
Kalantari et al. [54] Iran 2014	Cross sectional	FSGS	D	11	36	64	Urine	LC–MS	No data	Biomarkers Predict prognosis RNAS2 and Haptoglobin Pathways association with FSGS complement and coagulation
			DC							
			HC							
Ni et al. [55] China 2022	Longitudinal	FSGS	D Steroid sensitive	7	6	89	Biopsy	LC–MS	3131 to 4233 proteins 325 were differentially expressed between the Steroid sensitive and Steroid resistant group	Biomarkers Steroid resistant disease Lysosome associated membrane protein 1 (LAMP1) and Long chain fatty acyl-CoA synthetase 4 (ACSL4)
			D Steroid resistant fsgs	11	4	89				
			HC							
Chebotareva et al. [56] Russia 2022	Cross sectional	MCD FSGS	D MCD	9	38	51	Urine	LC–MS	76 proteins identified	C9, CD14 and SERPINA1 Biomarker for MCD AUC 0.893
			DC	30						
			HC							

^a^(PLA2R) Anti-phospholipase A2 receptor antibody add missing abbreviations

### Potential diagnostic biomarkers

The primary aim of 14 studies was to identify potential new biomarkers. Rood et al. [52] analysed urine samples in a small cohort of 5 MN patients and discovered LIMP-2 peptides (*p* < 0.01), a potential biomarker for Idiopathic MN compared to healthy controls and FSGS patients. Pang et al. [50] also compared a cohort of idiopathic MN, both Anti-phospholipase A2 receptor antibody- (PLA2R)negative (32) and positive (31), to healthy controls (32). Two potential biomarkers were highlighted, alpha-1-antitrypsin and afamin with follow up confirmation analysis performed using western blot analysis.

Samavat et al. [39] aimed to identify biomarkers for IgAN in urine samples of 13 patients alongside 8 healthy controls with an average age of 34 years. Ten proteins were either up-regulated or down-regulated compared to healthy controls, but no clear statistically significant biomarker was found. The outcome was similar for Xue et al. [40], again investigating IgAN using serum samples from 60 patients and 43 healthy controls, where 12 proteins were identified and validated but there were no statistically significant differences between groups to suggest a clear biomarker.

### Disease phenotype and risk of progression

Turnier et al. [46] carried out a paediatric study of LN using urine samples of 61 patients obtained within a month of renal biopsy. A potential biomarker was unveiled; α1-antichymotrypsin, encoded by the SERPINA3 gene, was found to have a moderate positive association (*p* = 0.005) with the histological disease severity measure National Institutes of Health Activity Index (NIH-AI). Mao et al. [49] performed proteomics on LN biopsy samples of 10 patients with an average age of 33 years. A previously researched protein, NEU1 [57], showed increased expression in patients with a higher chronicity index of disease and multivariate Cox regression analysis (HR, 6.462 (95% CI 1.025–40.732), *p* = 0.047) for renal prognosis. NEU1 was also found to be present in greater abundance in the urine samples of those patients.

Three further studies analysed a cohort of patients with IgAN. Paunas et al. [44] retrospectively analysed the biopsy samples of two cohorts of patients; 10 with no disease progression defined as no end-stage kidney disease (ESKD) 10 years post biopsy and 9 with ESKD 10 years post biopsy. Periostin showed promise as a novel and important risk marker of disease progression with AUC 0.91. Furthermore, stronger periostin staining by immunohistochemistry was subsequently seen in the progressive IgAN patients. Kalantari et al. [42] analysed urine samples from IgAN patients of different severity based on biopsy findings. Although no biomarkers were identified from 232 proteins, the results provided insight on the possible pathogenic pathways linked with disease progression. A further paediatric study by Fang et al. [41] investigated urine of 19 IgAN and 19 IgAV patients with an average age of 9 years and compared them to healthy controls. The metabolic pathways associated with the proteins identified were the complement and coagulation cascades and platelet activation. A1BG and AFM proteins were significantly increased in children with IgAN and IgAV but could not distinguish the two disease phenotypes.

### Treatment response

Three longitudinal studies applied proteomic analysis to establish a response to treatment. Ghasemi et al. [48] collected blood and urine samples from 19 LN patients at the time of renal biopsy. The patients were followed up for up to four years, the primary outcome being disease remission. Twenty plasma proteins and ten urine proteins could be identified as potential biomarkers.

Kalantari et al. [53] utilised urine samples from a cohort of 10 patients, six steroid-sensitive and four steroid-resistant, with FSGS at the time of biopsy. Steroid resistance was defined as failure to respond to the steroid regimen at eight weeks. Results showed a drastic fold change in two proteins, APOA-1 and MXRA8.

Ni et al. [55], carried out a paediatric study of 18 FSGS patients, 7 steroid-sensitive and 11 steroid-resistant, utilising biopsy samples with steroid resistance defined at six weeks. Two proteins, LAMP1 and ACSL4, previously described in the literature, were raised in steroid-resistant disease. These proteins were subsequently stained on biopsy samples to confirm their presence.

## Discussion

This review outlines the current utility of metabolomics and proteomics in children and adults with a histological diagnosis of GN. We aimed to establish the existing evidence and identify areas of unmet need. We reviewed 27 studies in total: 9 using metabolomic and lipidomic analysis, 18 using proteomics. The most frequently studied GN disease was IgAN, reflecting its place as the most prevalent primary glomerular disease worldwide [58]. Urine was the most frequently investigated sample type, and the study cohorts had an average cohort size of 113 in metabolomics and 35 in proteomics. The average age of participants was 35 years, and only four studies included a paediatric cohort.

The primary aim of most studies was to identify new diagnostic biomarkers. The aim is to produce less invasive and more rapid diagnostics alongside personalised medicine. To date, this area has been dominated by genomic discoveries in cancer [59, 60]. The most notable development in GN has been made in MN whereby Beck et al. [61] discovered a novel antibody M-type phospholipase A (2) receptor (PLA2R) using mass spectrometry, which is now widely used in clinical practice. This review highlighted several small-scale studies in nephrology; however, large studies were sparse. Tofte et al. [62] conducted a large multi-centre study of 1775 participants with type 2 diabetes and no proteinuria to validate the use of CKD273, a urinary biomarker composed of 273 peptides previously identified through proteomic analysis of CKD cohorts [63]. A scoring system was created based on CKD273 results that equate to the risk of developing proteinuria. This longitudinal study showed that patients with a high-risk score from the urinary biomarker CKD273 correlated with the development of proteinuria over a median of 2.5 years, independent of clinical characteristics [62].

Confirming disease activity and prognosis was another aim identified in this review applied to LN- and IgA-related nephropathy cohorts. In IgA patients, the aim was to identify urinary biomarkers that reflect the histological classification of disease and establish biomarkers from kidney histology that can correlate with predicting disease progression. IgAN has been researched using omic methods, and we have better insight into the pathogenesis and immunomodulatory changes that are key in this disease pattern. However, studies thus far have not yet succeeded in identifying biomarkers that can be utilised to develop precision nephrology and achieve personalised therapy [64, 65]. A recent study by Pitcher et al.[66] of long term outcomes in IgAN based on a UK registry showed a median (95% CI) kidney survival of 10.8 (10.0 to 12.0) years. At present, the role of immunosuppression is unclear in different disease phenotypes, and further omic analysis may introduce a better understanding of its benefits at certain stages of disease activity. Alonso et al. [67] analysed urinary metabolites using nuclear magnetic resonance across a range of autoimmune diseases including SLE and Crohn's. The results showed a clear pattern of metabolites that correlated with disease activity as per the currently used disease activity scores [68]. Without the advent of specific disease activity biomarkers, we are delaying the early initiation or alteration of treatment regimens that can affect the patients' long-term outcomes.

The mainstay of treatment in GN requires immunosuppression to achieve and sustain clinical remission. Whilst there are globally accepted guidelines for managing these diseases [28] there is still an unmet need in delivering personalised treatment to patients. Immunosuppressive regimens in GN are often dependent on glucocorticoids, which are increasingly highlighted as having significant long-term effects, including increasing the risk of cardiovascular events [69, 70]. The adoption of multi-omic data has led to the capture of a vast amount of data in cancer with the ability to predict prognosis and treatment response [71, 72]. However, this data's most successful progress and clinical adoption has been from genomic analysis. In prostate cancer, the development of the Decipher score has led to the use of genomic data to accurately predict which cancer will behave more aggressively and therefore inform treatment choices [73, 74]. Results from cancer genomics analyses have led to the creation of the 'Cancer Genome Atlas', which documents the molecular features of an array of cancers. This invaluable data tool can aid the classification of cancers and could represent a platform for further research to develop targeted treatment for specific cancer sub-types [75].

It has long been established that cardiovascular disease is the leading cause of mortality in individuals diagnosed with CKD [76–79]. To date, we are continuing to unravel the interplay of disease processes and sociodemographic risk factors that accelerate atherosclerosis in CKD populations and the unique pro-inflammatory states associated [80, 81]. Lipidomic analysis has successfully shown that changes within the lipidome interplay and contribute to the pro-inflammatory state leading to atherosclerosis in cardiovascular disease and atherosclerosis in CKD [82, 83].

The main limitation of the included studies is the sample size, especially in proteomic analysis, where the average cohort size was only 35. To produce statistically significant results and uncover potential new biomarkers, these studies should aim to recruit larger cohorts. Multiple tools have been developed to support the multi-omic analysis power calculation for multi-omic analysis [84]. The technology needed for multi-omic analysis requires expensive infrastructure, which may not be so widely accessible, with finances limiting the sample size. The papers were heterogeneous in their methodology and data analysis, making direct comparison of their outcomes and significance more challenging. Moreover, only four studies included paediatric patients, perhaps representing cohorts with a more active phenotype and fewer co-morbidities.

## Conclusion

This review details the current metabolomic and lipidomic analysis landscape in GN. There is clear evidence that the application of omic techniques through the analysis of blood, urine and kidney histology can elucidate the immunopathogenesis of GN and contribute to the development of precision medicine in nephrology.

## References

[1] Kovesdy CP (2022) Epidemiology of chronic kidney disease: an update 2022. Kidney Int Suppl (2011) 12(1):7–11 35529086 10.1016/j.kisu.2021.11.003 PMC9073222

[2] Bikbov B et al (2020) Global, regional, and national burden of chronic kidney disease, 1990–2017: a systematic analysis for the global burden of disease study 2017. Lancet 395(10225):709–733 32061315 10.1016/S0140-6736(20)30045-3 PMC7049905

[3] Carney EF (2020) The impact of chronic kidney disease on global health. Nat Rev Nephrol 16(5):251 32144399 10.1038/s41581-020-0268-7

[4] Nissenson AR et al (2001) Opportunities for improving the care of patients with chronic renal insufficiency: current practice patterns. J Am Soc Nephrol 12(8):1713–1720 11461944 10.1681/ASN.V1281713

[5] Ma I et al (2018) Sociodemographic associations with abnormal estimated glomerular filtration rate (eGFR) in a large Canadian city: a cross-sectional observation study. BMC Nephrol 19(1):198 30092764 10.1186/s12882-018-0991-5 PMC6085713

[6] Stevens PE et al (2007) Chronic kidney disease management in the United Kingdom: NEOERICA project results. Kidney Int 72(1):92–99 17440495 10.1038/sj.ki.5002273

[7] Hu J et al (2023) Global, regional, and national burden of CKD due to glomerulonephritis from 1990 to 2019: a systematic analysis from the global burden of disease study 2019. Clin J Am Soc Nephrol 18(1):60–71 36719159 10.2215/CJN.0000000000000017 PMC10101559

[8] Registry, U.R. UK Renal Registry (2022) UK renal registry 24th annual report—data to 31/12/2020. 2022. Accessed 06 Aug 2023]. Available from: https://ukkidney.org/audit-research/annual-report.

[9] Chadban SJ, Atkins RC (2005) Glomerulonephritis. Lancet 365(9473):1797–1806 15910953 10.1016/S0140-6736(05)66583-X

[10] Anders H-J, Jayne DRW, Rovin BH (2016) Hurdles to the introduction of new therapies for immune-mediated kidney diseases. Nat Rev Nephrol 12(4):205–216 26804020 10.1038/nrneph.2015.206

[11] Anders HJ et al (2023) Glomerulonephritis: immunopathogenesis and immunotherapy. Nat Rev Immunol 23(7):453–471 36635359 10.1038/s41577-022-00816-y PMC9838307

[12] Hartl D et al (2021) Translational precision medicine: an industry perspective. J Transl Med 19(1):245 34090480 10.1186/s12967-021-02910-6 PMC8179706

[13] Subramanian I et al (2020) Multi-omics data integration, interpretation, and its application. Bioinform Biol Insights 14:1177932219899051 32076369 10.1177/1177932219899051 PMC7003173

[14] Schmidt DR et al (2021) Metabolomics in cancer research and emerging applications in clinical oncology. CA Cancer J Clin 71(4):333–358 33982817 10.3322/caac.21670 PMC8298088

[15] Babu M, Snyder M (2023) Multi-omics profiling for health. Mol Cell Proteomics 22(6):100561 37119971 10.1016/j.mcpro.2023.100561 PMC10220275

[16] Yamada R et al (2021) Interpretation of omics data analyses. J Hum Genet 66(1):93–102 32385339 10.1038/s10038-020-0763-5 PMC7728595

[17] Kang M, Ko E, Mersha TB (2022) A roadmap for multi-omics data integration using deep learning. Brief Bioinform 23(1):bbab454 34791014 10.1093/bib/bbab454 PMC8769688

[18] Verrills NM (2006) Clinical proteomics: present and future prospects. Clin Biochem Rev 27(2):99–116 17077880 PMC1579414

[19] Dotz V et al (2021) O- and N-glycosylation of serum immunoglobulin a is associated with iga nephropathy and glomerular function. J Am Soc Nephrol 32(10):2455–2465 34127537 10.1681/ASN.2020081208 PMC8722783

[20] Kell DB et al (2005) Metabolic footprinting and systems biology: the medium is the message. Nat Rev Microbiol 3(7):557–565 15953932 10.1038/nrmicro1177

[21] Zhao Y-Y (2013) Metabolomics in chronic kidney disease. Clin Chim Acta 422:59–69 23570820 10.1016/j.cca.2013.03.033

[22] Chetwynd AJ et al (2017) Nanoflow-nanospray mass spectrometry metabolomics reveals disruption of the urinary metabolite profiles of HIV-positive patients on combination antiretroviral therapy. JAIDS J Acquir Immune Defic Syndr 74(2):e45-53 27552076 10.1097/QAI.0000000000001159

[23] Guida F et al (2021) The blood metabolome of incident kidney cancer: a case-control study nested within the MetKid consortium. PLoS Med 18(9):e1003786 34543281 10.1371/journal.pmed.1003786 PMC8496779

[24] Han X, Gross RW (2022) The foundations and development of lipidomics. J Lipid Res 63(2):100164 34953866 10.1016/j.jlr.2021.100164 PMC8953652

[25] Baek J et al (2022) Lipidomic approaches to dissect dysregulated lipid metabolism in kidney disease. Nat Rev Nephrol 18(1):38–55 34616096 10.1038/s41581-021-00488-2 PMC9146017

[26] Eddy S, Mariani LH, Kretzler M (2020) Integrated multi-omics approaches to improve classification of chronic kidney disease. Nat Rev Nephrol 16(11):657–668 32424281 10.1038/s41581-020-0286-5

[27] Provenzano M et al (2021) OMICS in chronic kidney disease: focus on prognosis and prediction. Int J Mol Sci 23(1):336 35008760 10.3390/ijms23010336 PMC8745343

[28] Rovin BH et al (2021) Executive summary of the KDIGO 2021 guideline for the management of glomerular diseases. Kidney Int 100(4):753–779 34556300 10.1016/j.kint.2021.05.015

[29] Programme, C.A.S. CASP Cohort study Checklist. 2002. Accessed 06 aug 2023. Available from: https://casp-uk.net/images/checklist/documents/CASP-Cohort-Study-Checklist/CASP-Cohort-Study-Checklist-2018_fillable_form.pdf

[30] Zhang Q et al (2021) Serum-urine matched metabolomics for predicting progression of henoch-schonlein purpura nephritis. Front Med. 10.3389/fmed.2021.657073 34055834 10.3389/fmed.2021.657073 PMC8149729

[31] Kalantari S et al (2017) 1 H NMR-based metabolomics study for identifying urinary biomarkers and perturbed metabolic pathways associated with severity of IgA nephropathy: a pilot study. Magn Reson Chem 55(8):693–699 28042675 10.1002/mrc.4573

[32] Park S et al (2021) Comprehensive metabolomic profiling in early IgA nephropathy patients reveals urine glycine as a prognostic biomarker. J Cell Mol Med 25(11):5177–5190 33939273 10.1111/jcmm.16520 PMC8178259

[33] Guleria A et al (2016) NMR based serum metabolomics reveals a distinctive signature in patients with Lupus Nephritis. Sci Rep 6:35309 27739464 10.1038/srep35309 PMC5064370

[34] Kalantari S et al (2019) Metabolomics approach reveals urine biomarkers and pathways associated with the pathogenesis of lupus nephritis. Iran J Basic Med Sci 22(11):1288–1295 32128093 10.22038/ijbms.2019.38713.9178 PMC7038420

[35] Taherkhani A et al (2019) Metabolomic analysis of membranous glomerulonephritis: identification of a diagnostic panel and pathogenic pathways. Arch Med Res 50(4):159–169 31499475 10.1016/j.arcmed.2019.08.004

[36] Hao X et al (2013) Distinct metabolic profile of primary focal segmental glomerulosclerosis revealed by NMR-based metabolomics. PLoS ONE 8(11):e78531 24244321 10.1371/journal.pone.0078531 PMC3823857

[37] Erkan E et al (2016) Distinct urinary lipid profile in children with focal segmental glomerulosclerosis. Pediatr Nephrol 31(4):581–588 26537928 10.1007/s00467-015-3239-7 PMC4962780

[38] Lee JE et al (2016) Systematic biomarker discovery and coordinative validation for different primary nephrotic syndromes using gas chromatography-mass spectrometry. J Chromatogr A 1453:105–115 27247212 10.1016/j.chroma.2016.05.058

[39] Samavat S et al (2015) Diagnostic urinary proteome profile for immunoglobulin a nephropathy. Iran J Kidney Dis 9(3):239–248 25957429

[40] Xue D et al (2023) Serum proteomic analysis by nanoflow LC-MS/MS-based proteomics in iga chronic kidney disease. Clin Lab 69(3):01 10.7754/Clin.Lab.2022.220417 36912316

[41] Fang X et al (2021) Use of liquid chromatography-tandem mass spectrometry to perform urinary proteomic analysis of children with IgA nephropathy and Henoch-Schonlein purpura nephritis. J Proteomics 230:103979 32932007 10.1016/j.jprot.2020.103979

[42] Kalantari S et al (2013) Urinary prognostic biomarkers and classification of IgA nephropathy by high resolution mass spectrometry coupled with liquid chromatography. PLoS ONE [Electronic Resource] 8(12):e80830 24339887 10.1371/journal.pone.0080830 PMC3855054

[43] Mucha K et al (2014) Complement components, proteolysis-related, and cell communication-related proteins detected in urine proteomics are associated with IgA nephropathy. Pol Arch Med Wewn 124(7):380–386 24938435 10.20452/pamw.2345

[44] Paunas FTI et al (2022) Proteomic signature of tubulointerstitial tissue predicts prognosis in IgAN. BMC Nephrol 23(1):118 35331167 10.1186/s12882-022-02736-4 PMC8943973

[45] Kawata N et al (2020) Proteomics of human glomerulonephritis by laser microdissection and liquid chromatography-tandem mass spectrometry. Nephrology 25(4):351–359 31707756 10.1111/nep.13676 PMC7064884

[46] Turnier JL et al (2019) Discovery of SERPINA3 as a candidate urinary biomarker of lupus nephritis activity. Rheumatology 58(2):321–330 30285245 10.1093/rheumatology/key301 PMC6343468

[47] Chen YY et al (2022) Proteomic profiling of kidney samples in patients with pure membranous and proliferative lupus nephritis. Lupus 31(7):837–847 35446734 10.1177/09612033221094711

[48] Ghasemi M et al (2021) Predictive biomarker panel in proliferative lupus nephritis- two-dimensional shotgun proteomics. Iran J Kidney Dis 1(2):121–133 33764323

[49] Mao Z et al (2021) Discovery of NEU1 as a candidatedone. renal biomarker for proliferative lupus nephritis chronicity. Lupus Sci Med 8(1):12 10.1136/lupus-2021-000569 PMC8650488 34872988

[50] Pang L et al (2018) Urine proteomics of primary membranous nephropathy using nanoscale liquid chromatography tandem mass spectrometry analysis. Clin Proteom 15(1):1–15 10.1186/s12014-018-9183-3 PMC5801694 29445323

[51] Li S et al (2022) Label-free quantitative proteomic and phosphoproteomic analyses of renal biopsy tissues in membranous nephropathy. Proteomics Clin Appl 16(1):e2000069 34543527 10.1002/prca.202000069

[52] Rood IM et al (2015) Increased expression of lysosome membrane protein 2 in glomeruli of patients with idiopathic membranous nephropathy. Proteomics 15(21):3722–3730 26304790 10.1002/pmic.201500127

[53] Kalantari S et al (2014) Predictive urinary biomarkers for steroid-resistant and steroid-sensitive focal segmental glomerulosclerosis using high resolution mass spectrometry and multivariate statistical analysis. BMC Nephrol 15:141 25182141 10.1186/1471-2369-15-141 PMC4236676

[54] Kalantari S et al (2014) Urinary prognostic biomarkers in patients with focal segmental glomerulosclerosis. Nephro-Urology Mon 6(2):e16806 10.5812/numonthly.16806 PMC4090581 25032130

[55] Ni J et al (2022) Comparative proteomic analysis of children FSGS FFPE tissues. BMC Pediatr 22(1):707 36503536 10.1186/s12887-022-03764-7 PMC9743561

[56] Chebotareva NV et al (2022) Potential urine proteomic biomarkers for focal segmental glomerulosclerosis and minimal change disease. Int J Mol Sci 23(20):20 10.3390/ijms232012607 PMC9604469 36293475

[57] Nowling TK et al (2015) Renal glycosphingolipid metabolism is dysfunctional in lupus nephritis. J Am Soc Nephrol 26(6):1402–1413 25270066 10.1681/ASN.2014050508 PMC4446878

[58] Schena FP, Nistor I (2018) Epidemiology of IgA nephropathy: a global perspective. Semin Nephrol 38(5):435–442 30177015 10.1016/j.semnephrol.2018.05.013

[59] Chen G et al (2023) Integrative analysis of multi-omics data for liquid biopsy. Br J Cancer 128(4):505–518 36357703 10.1038/s41416-022-02048-2 PMC9938261

[60] Alix-Panabières C, Pantel K (2021) Liquid biopsy: from discovery to clinical application. Cancer Discov 11(4):858–873 33811121 10.1158/2159-8290.CD-20-1311

[61] Beck LH Jr et al (2009) M-type phospholipase A2 receptor as target antigen in idiopathic membranous nephropathy. N Engl J Med 361(1):11–21 19571279 10.1056/NEJMoa0810457 PMC2762083

[62] Tofte N et al (2020) Early detection of diabetic kidney disease by urinary proteomics and subsequent intervention with spironolactone to delay progression (PRIORITY): a prospective observational study and embedded randomised placebo-controlled trial. Lancet Diabetes Endocrinol 8(4):301–312 32135136 10.1016/S2213-8587(20)30026-7

[63] Good DM et al (2010) Naturally occurring human urinary peptides for use in diagnosis of chronic kidney disease. Mol Cell Proteomics 9(11):2424–2437 20616184 10.1074/mcp.M110.001917 PMC2984241

[64] Schena FP et al (2018) Omics studies for comprehensive understanding of immunoglobulin A nephropathy: state-of-the-art and future directions. Nephrol Dial Transplant 33(12):2101–2112 29905852 10.1093/ndt/gfy130

[65] Mucha K, Pac M, Pączek L (2023) Omics are getting Us closer to understanding IgA nephropathy. Arch Immunol Ther Exp (Warsz) 71(1):12 37060455 10.1007/s00005-023-00677-w PMC10105675

[66] Pitcher D et al (2023) Long-term outcomes in IgA nephropathy. Clin J Am Soc Nephrol 18(6):727–738 37055195 10.2215/CJN.0000000000000135 PMC10278810

[67] Alonso A et al (2016) Urine metabolome profiling of immune-mediated inflammatory diseases. BMC Med 14(1):133 27609333 10.1186/s12916-016-0681-8 PMC5016926

[68] Ceccarelli F et al (2015) Assessment of disease activity in systemic lupus erythematosus: lights and shadows. Autoimmun Rev 14(7):601–608 25742757 10.1016/j.autrev.2015.02.008

[69] Huscher D et al (2009) Dose-related patterns of glucocorticoid-induced side effects. Ann Rheum Dis 68(7):1119–1124 18684744 10.1136/ard.2008.092163

[70] Moya FB, Galindo LFP, de la Peña MG (2016) Impact of chronic glucocorticoid treatment on cardiovascular risk profile in patients with systemic lupus erythematosus. J Clin Rheumatol 22(1):8–12 26693620 10.1097/RHU.0000000000000335

[71] Olivier M et al (2019) The need for multi-omics biomarker signatures in precision medicine. Int J Mol Sci 20(19):4781 31561483 10.3390/ijms20194781 PMC6801754

[72] Shmatko A et al (2022) Artificial intelligence in histopathology: enhancing cancer research and clinical oncology. Nature Cancer 3(9):1026–1038 36138135 10.1038/s43018-022-00436-4

[73] Erho N et al (2013) Discovery and validation of a prostate cancer genomic classifier that predicts early metastasis following radical prostatectomy. PLoS ONE 8(6):e66855 23826159 10.1371/journal.pone.0066855 PMC3691249

[74] Jairath NK et al (2021) A systematic review of the evidence for the decipher genomic classifier in prostate cancer. Eur Urol 79(3):374–383 33293078 10.1016/j.eururo.2020.11.021

[75] Hutter C, Zenklusen JC (2018) The cancer genome atlas: creating lasting value beyond its data. Cell 173(2):283–285 29625045 10.1016/j.cell.2018.03.042

[76] Jankowski J et al (2021) Cardiovascular disease in chronic kidney disease: pathophysiological insights and therapeutic options. Circulation 143(11):1157–1172 33720773 10.1161/CIRCULATIONAHA.120.050686 PMC7969169

[77] Matsushita K et al (2022) Epidemiology and risk of cardiovascular disease in populations with chronic kidney disease. Nat Rev Nephrol 18(11):696–707 36104509 10.1038/s41581-022-00616-6

[78] Matsushita K et al (2015) Estimated glomerular filtration rate and albuminuria for prediction of cardiovascular outcomes: a collaborative meta-analysis of individual participant data. Lancet Diabetes Endocrinol 3(7):514–525 26028594 10.1016/S2213-8587(15)00040-6 PMC4594193

[79] Sarnak MJ et al (2003) Kidney disease as a risk factor for development of cardiovascular disease. Circulation 108(17):2154–2169 14581387 10.1161/01.CIR.0000095676.90936.80

[80] Balla S, Nusair MB, Alpert MA (2013) Risk factors for atherosclerosis in patients with chronic kidney disease: recognition and management. Curr Opin Pharmacol 13(2):192–199 23291030 10.1016/j.coph.2012.12.001

[81] Valdivielso JM et al (2019) Atherosclerosis in chronic kidney disease: more, less, or just different? Arterioscler Thromb Vasc Biol 39(10):1938–1966 31412740 10.1161/ATVBAHA.119.312705

[82] Ding M, Rexrode KM (2020) A review of lipidomics of cardiovascular disease highlights the importance of isolating lipoproteins. Metabolites 10(4):163 32340170 10.3390/metabo10040163 PMC7240942

[83] Tracz J, Luczak M (2021) Applying proteomics and integrative “Omics” strategies to decipher the chronic kidney disease-related atherosclerosis. Int J Mol Sci 22(14):7492 34299112 10.3390/ijms22147492 PMC8305100

[84] Tarazona S et al (2020) Harmonization of quality metrics and power calculation in multi-omic studies. Nat Commun 11(1):3092 32555183 10.1038/s41467-020-16937-8 PMC7303201

